# Unravelling the associations between local environmental factors, soil properties and cultivable root-associated endophytes in dry pea (*Pisum sativum* L.)

**DOI:** 10.1007/s11274-026-05092-9

**Published:** 2026-06-22

**Authors:** Shiying Qu, Jorge Martín-García, Daniel Martín-Jiménez, Irene Zunzunegui, Tamara Sánchez-Gómez, Óscar Santamaría, Jorge Poveda

**Affiliations:** Department of Plant Production and Forest Resources, Higher Technical School of Agricultural Engineering of Palencia, Recognised Research Group AGROBIOTECH, UIC-370 (JCyL), University Institute for Research in Sustainable Forest Management (iuFOR), University of Valladolid, Avda. Madrid 57, Palencia, 34004 Spain

**Keywords:** *Pisum sativum*, Root endophytes, Cultivable microbiome, Soil texture, Exchangeable magnesium

## Abstract

Endophytic microorganisms in legume roots form an important interface between soil conditions and plant health. However, field-scale evidence remains limited on how cultivable endophytic bacterial and fungal assemblages in pea roots vary with soil and climatic conditions after nodule removal. In this study, 96 healthy pea plants were collected from eight field plots in Palencia, Spain. Cultivable endophytic bacteria and fungi were isolated from surface-disinfected root tissues after nodule removal, and community composition was assessed by Sanger sequencing of 16 S rRNA gene and ITS markers using a 97% OTU workflow. Bacterial communities showed greater compositional variation among plots, although genera such as *Pseudomonas*, *Achromobacter*, *Peribacillus* and *Bacillus* were repeatedly isolated across sites. In the cultivable fungal fraction, *Fusarium* was the most frequently isolated genus. Non-metric multidimensional scaling (NMDS)-envfit analysis showed that bacterial community variation was significantly associated with soil silt percentage (r² = 0.87, *P* = 0.007), whereas fungal community variation was significantly associated with exchangeable magnesium (r² = 0.67, *P* = 0.046). No climatic variable showed a significant fit at the study scale. Overall, these results provide a culture-based description of recurrent endophytic taxa in healthy pea roots and their preliminary associations with local soil conditions, offering a basis for future strain-based functional studies and more comprehensive microbiome analyses using culture-independent approaches.

**Supplementary Information:**

The online version contains supplementary material available at 10.1007/s11274-026-05092-9.

## Introduction

Endophytic microorganisms, defined as microbes that inhabit plant tissues without inducing visible disease symptoms, constitute an important component of the plant-microbe symbiotic system. They contribute to host fitness by stimulating plant growth, strengthening resistance against pathogens, and improving nutrient uptake efficiency (Poveda et al. 2022; Teiba et al. 2023). As such, they are increasingly recognized as key contributors to environmentally sustainable agricultural systems (Chauhan et al. 2023). In recent years, with the advancement of microbial ecology, the diversity and functional significance of plant endophytes have garnered growing scientific interest (Minchev et al. 2024).

Among various crop types, legumes are particularly important due to their nitrogen-fixing capacity and high protein content, making them essential components of agricultural ecosystems (Thavarajah et al. 2023). *Pisum sativum* L. (dried peas), as a representative legume crop, is widely consumed in human diets and serves as a valuable protein-rich feed for ruminants (Thavarajah et al. 2023; Wu et al. 2023). Agronomically, it has widely been used as a rotational crop, due to its capacity for improving soil fertility and structure within sustainable farming systems (Smýkal et al. 2020). In addition, peas might have a high grain yield under rainfed conditions because of their drought and diseases tolerance. For all these reasons, it is the most cultivated grain legume in Europe with more than 1 M ha (Food and Agriculture Organization of the United Nations, 2024). In temperate regions, such as Castilla y León (Northwest Spain), a major agricultural area characterized by continental climate, diverse soil types, and variable land management practices, where dry pea is extensively cultivated, its grain yield ranges between 700 and 1,900 kg ha^− 1^ (MAPA, 2025). The strong variability in productivity has been found to depend mainly on the cultivar used (Kadžiulienė et al. 2025) and the environmental conditions (Uhlarik et al. 2024). The cropping system also affects productivity according to several studies (Iivonen et al. 2024), where organic pea yields averaged about 65% of conventional yields, although with substantial potential for improvement.

Soil microbiota, as well as that inhabiting the crop root system, is a crucial factor to improve growth and hence grain yield, not only in organic but also in conventional and integrated production systems (Pandey and Saharan 2025; Watts et al. 2023). Therefore, knowing the microbial communities occurring in the crop rhizosphere and the factors affecting their performance might be a key aspect to use these organisms more efficiently in a more environmentally friendly agriculture. There are several studies in which the effect of environmental variables, such as soil physicochemical properties and climate, on the root microbiome has been assessed (Richardson et al. 2021). Even in peas, the role of water stress, cadmium, and plant genotype has been investigated on the rhizosphere microbiome (Kichko et al. 2022). However, most of existing studies on plant-associated microbes focus on diseased or stressed plants or target specific microbial groups, such as mycorrhizal fungi or rhizobial bacteria (Liu et al. 2024; Compant et al. 2025). Systematic investigations into the full endophytic communities in the roots of healthy dried peas remain limited. Pea roots are colonized by a taxonomically diverse microbiota, including the nitrogen-fixing rhizobia housed within root nodules as well as a broad range of non-rhizobial bacterial and fungal endophytes distributed throughout the root tissues (Tariq et al. 2014; Mayhood and Mirza 2021). Because the rhizobial symbionts concentrated in nodules are already comparatively well characterized and would otherwise dominate the recovered isolates, nodules were excised prior to isolation so that the present study could focus on the broader and less-studied endophytic community inhabiting the root tissues themselves.

To address these gaps, this study focuses on healthy pea root systems to (i) describe the composition and diversity characteristics of cultivable endophytic microorganisms across different sites (ii) compare changes in community composition under varying environmental conditions, and (iii) explore potential associations between community differences and soil physicochemical properties as well as local climatic factors. This study provides foundational data for understanding the ecological distribution characteristics of cultivable endophytic microorganisms in healthy pea root systems, and to offer a reference for subsequent screening of functional strains and the conduct of more in-depth crop microbiome research in conjunction with high-throughput molecular methods.

## Materials and methods

### Site characteristics and sampling

Sampling was conducted in eight plots located in the province of Palencia (Northwest Spain). All plots were sown with dried peas in winter 2024, following barley (*Hordeum vulgare* L.) as the previous crop. Sowing took place between 25 January and 4 February 2024. Based on field records about soil tillage and sowing method (Supplementary Table 1), plots were assigned to one of the following three categories (Table 1) for the subsequent statistical analysis: Category 1: Surface work (Plots 1–4), harrowing and conventional sowing with jet seed drills; Category 2: Direct seeding (Plots 5–6), with no tillage and no-till seed drills; and Category 3: Intermediate work (Plots 7–8), pre-sowing soil preparation including mini-chisel, disc harrowing, and rolling and sowing with jet seed drills. The complete details of the cropping activities followed in each plot are given in Supplementary Table 1, which are summarized and standardized, for statistical purposes, in Table 1.

**Table 1:** Summary of field management categorical variables for eight plots

Plot	Sowing date (days)	Work tasks	Herbicide	Insecticide	Fertilized
Plot1	8	Surface work	yes	no	no
Plot2	8	Surface work	yes	no	no
Plot3	8	Surface work	yes	no	no
Plot4	8	Surface work	yes	no	no
Plot5	0	Direct seeding	yes	no	no
Plot6	0	Direct seeding	yes	no	no
Plot7	9	Intermediate work	yes	yes	yes
Plot8	10	Intermediate work	yes	yes	yes

Note: Sowing date is expressed as days since the earliest sowing (0 = 25th January 2024). Work tasks summarize establishment operations into three categories: Surface work (involving only surface or shallow operations such as harrowing or rolling), Direct seeding (sowing without prior land preparation), and Intermediate work (involving at least one moderate-intensity tillage or land preparation operation prior to sowing, such as chiselling or disc harrowing). More details about plots cropping systems are provided in Supplementary Table 1

Each plot was divided into four zones separated by at least 8 m. In each zone, three apparently healthy pea plants were carefully uprooted, giving a total of 96 plant samples (12 plants per plot). Each plant was placed in a sterile, hermetically sealed plastic bag to prevent cross-contamination, transported under refrigerated conditions (4 °C), and processed within 24 h for microbiological analysis. For each plot, the bacterial and fungal isolates obtained from all 12 plants were pooled and treated as a single plot-level sample, so that all subsequent community composition and diversity analyses were conducted at the plot level.

Soil samples were collected from the same zones at a depth of 10–30 cm, corresponding to the active root zone of pea plants. Two composite soil samples, each weighing approximately 1 kg, were obtained per plot by homogenising subsamples from two adjacent zones, resulting in a total of 16 composite soil samples. Soil pH was determined in a 1:2.5 soil-to-water suspension using a calibrated pH meter (potentiometry). Electrical conductivity (EC) was measured in a 1:5 soil-to-water extract using a conductivity meter. Particle-size distribution was determined by the Bouyoucos method (hydrometer method), and the ISSS (International Society of Soil Science) classification was applied. Oxidizable organic matter was measured by the K₂Cr₂O₇ method. Total N was determined by the modified Kjeldahl method. Available P was measured by the Olsen–NaHCO₃ method. Soil available K and exchangeable Ca^2+^, Mg^2+^, and Na^2+^ were extracted with ammonium acetate and determined by inductively coupled plasma–optical emission spectrometry (ICP-OES). For each plot, results from the two composite soil samples were summarized as mean with standard error (SE) and used for subsequent analyses (Supplementary Table 2). The predominant soil texture is sandy loam, with some plots classified as sandy clay or clay. Soil pH values range from neutral to slightly alkaline (7.9–8.6). Soil organic matter and total nitrogen contents were generally low across all plots (typically below 2% and 0.15% respectively), with Plot 7 exhibiting the highest values (organic matter: 3.83%; total nitrogen: 0.275%). Readily available phosphorus varied substantially among plots (< 5 to 60.65 mg kg^− 1^).

Climate data were obtained from the Digital Climatic Atlas of the Iberian Peninsula (Ninyerola et al. 2005). For each plot, we extracted Universal Transverse Mercator (UTM) coordinates, total and mean precipitation, minimum temperature, maximum temperature and mean temperature with SE (spring, summer, autumn winter and annual). Annual precipitation ranged from 405.8 to 470.7 millimetres, while the annual mean temperature ranged from 11.5 to 11.9 °C. Seasonal precipitation is relatively evenly distributed across spring, autumn and winter, whereas summer precipitation is comparatively low (Supplementary Table 2).

### Isolation of endophytic bacteria and fungi

Root samples with nodules excised were subjected to surface sterilization following a modified protocol adapted from Kumar et al. (2023). Roots were immersed in 70% ethanol for 30 s, followed by 2.5% sodium hypochlorite for 3 min, and finally rinsed three times with sterile distilled water. To verify the effectiveness of sterilization, aliquots of the last rinse water were poured onto tryptic soy agar (TSA, PanReac AppliChem, 40 g/L) and potato dextrose agar (PDA, Scharlab, 39 g/L) plates and incubated to check for microbial growth.

For fungal isolation, 1 cm segments of surface-sterilized roots were placed onto PDA supplemented with chloramphenicol (Sigma-Aldrich, 200 mg/L), with five replicates per plant sample. Plates were incubated at 25 ± 2 °C in the dark for up to 14 days. For bacterial isolation, sterilized root tissues were macerated in sterile distilled water and streaked onto TSA plates, which were incubated at 28 ± 2 °C until colonies developed. Emerging colonies with distinct morphologies were sub-cultured repeatedly to obtain pure isolates.

Morphologically distinct colonies were grouped into morphotypes based on macroscopic characteristics (color, margin, elevation, texture and growth rate). From each morphotype, one or two representative isolates were selected for molecular identification, under the assumption that the sequence obtained was representative of the entire morphotype.

Purified fungal isolates were maintained on agar medium and stored at 4 °C as working cultures for subsequent handling and confirmation, when needed. In parallel, bacterial isolates were cultured in Luria–Bertani Broth (LB, Sigma-Aldrich, 20 g/L) to the logarithmic growth phase. Aliquots were mixed with sterile glycerol (final concentration 25%, v/v) and archived at − 80 °C.

### Molecular analysis and sequence processing

Genomic DNA was extracted from fungal mycelia and bacterial colonies using an Extract DNA Kit (Thermo Fisher Scientific Baltics UAB, Lithuania) following the manufacturer’s instructions. For bacteria, nearly full-length 16S rRNA genes were amplified using the universal forward primer P1 (5’-CGGGATCCAGAGTTTGATCCTGGTCAGA ACGAACGCT-3’) and reverse primer P6 (5’-CGGGATCCT ACGGCTACCTTGTTACGACTTCACCCC-3’) (Tariq et al. 2014), and amplicons were sequenced by single-end Sanger sequencing. For fungi, the internal transcribed spacer (ITS) region was amplified using primers ITS1 (5’-TCCGTAGGTGAACCTGCGG-3’) and ITS4 (5’-TCCTCCGCTTATTGATATGC-3’) (White et al. 1990), also sequenced by single-end Sanger sequencing.

Raw sequences were processed in QIIME2 v2025.7, including trimming of low-quality bases (Phred score < 20–30), removal of primer remnants and ambiguous bases (“N”), and exclusion of short or low-quality reads. High-quality sequences were dereplicated with VSEARCH v2.22.1, and potential chimeras were removed using the uchime ref algorithm. Non-chimeric sequences were clustered into Operational Taxonomic Units (OTUs) at 97% similarity using centroid-based clustering, yielding representative centroid sequences and OTU membership information.

Taxonomic assignments were performed on centroid sequences using BLAST+ v2.14.1 against the SILVA r138.1 database for bacteria and the UNITE v9.0 (April 2023 release, dynamic set) for fungi. Annotations were retained at the genus level when alignments reached ≥ 97% sequence identity and ≥ 90% query coverage, and at the order level when sequence identity was between 94% and 97%. Curated centroid sequences were deposited in NCBI GenBank, and OTU-level accession numbers and taxonomic details are summarized in Table 2 (endophytic bacteria) and Table 3 (endophytic fungi).

**Table 2:** Isolation frequencies of endophytic bacterial OTUs (operational taxonomic units) (indicating the relative frequency, %, in brackets) in relation to the plots and tissues from which they were recovered, Palencia, northern Spain

OTUs	Genbank Acc no.	Best hit (Genbank Acc no.)	qcovs	evalue	Plot 1	Plot 2	Plot 3	Plot 4	Plot 5	Plot 6	Plot 7	Plot 8	Total
BacID30	PV872195	*Cytobacillus* sp (KY038814.1)	92	0	1 (1.45)								1 (0.16)
BacID36	PV872201	*Pseudomonas* sp2 (AB680222.1)	100	0	30 (43.48)		5 (4.63)	11 (15.71)	20 (20.83)	31 (38.75)	10 (11.90)		107 (16.80)
BacID25	PV872190	*Achromobacter* sp2 (CP025030.)	100	0	15 (21.74)		8 (7.41)	10 (14.29)		12 (15.00)	2 (2.38)		47 (7.38)
BacID119	PV872284	*Peribacillus* sp2 (AB680229.1)	100	0		7 (15.91)	3 (2.78)		50 (52.08)	34 (42.50)	39 (46.43)	15 (17.44)	148 (23.23)
BacID1	PV872166	*Brevibacillus* sp (AB680946.1)	100	0			2 (1.85)				5 (5.95)		7 (1.10)
BacID15	PV872180	*Bacillus* sp3 (FBXX01000012)	100	0	4 (5.80)								4 (0.63)
BacID19	PV872184	*Bacillus* sp4 (CP011007)	100	0	2 (2.90)						7 (8.33)		9 (1.41)
BacID134	PV872299	*Bacillus* sp5 (AAJM01000140)	100	0			7 (6.48)			2 (2.50)	15 (17.86)		24 (3.77)
BacID4	PV872169	*Lysinibacillus* sp2 (AB330395.1)	100	0			1 (0.93)						1 (0.16)
BacID116	PV872281	*Exiguobacterium* sp (AB680657.1)	100	0			1 (0.93)		2 (2.08)				3 (0.47)
BacID160	PV872325	*Peribacillus* sp1 (CP011008)	100	0	15 (21.74)	4 (9.09)	40 (37.04)	39 (55.71)	22 (22.92)	1 (1.25)		54 (62.79)	175 (27.47)
BacID173	PV872338	*Bacillus* sp1 (AAJM01000140)	100	0			24 (22.22)				1 (1.19)	11 (12.79)	36 (5.65)
BacID143	PV872308	*Paenibacillus* sp1 (CP011420)	100	0			3 (2.78)				3 (3.57)		6 (0.94)
BacID163	PV872328	Lysinibacillus sp1 (AF169527.1)	100	0			3 (2.78)						3 (0.47)
BacID67	PV872232	*Achromobacter* sp1 (HE613447.1)	100	0	2 (2.90)	33 (75.00)	9 (8.33)	5 (7.14)					49 (7.69)
BacID159	PV872324	*Paenibacillus* sp2 (CP018620)	100	0					2 (2.08)				2 (0.31)
BacID181	PV872346	*Paenibacillus* sp3 (AB073201.1)	100	0								5 (5.81)	5 (0.78)
BacID69	PV872234	*Paenibacillus* sp4 (FN677987.1)	100	0			1 (0.93)						1 (0.16)
BacID158	PV872323	*Pseudomonas* sp1 (AKJR01000012)	100	0			1 (0.93)				2 (2.38)		3 (0.47)
BacID168	PV872333	*Bacillus* sp2 (CP007800)	100	0								1 (1.16)	1 (0.16)
BacID98	PV872263	*Luteibacter* sp (AY785744.1)	100	1.85E-140				5 (7.14)					5 (0.78)
Total					69	44	108	70	96	80	84	86	637

Note: Values indicate the number of isolates, with relative frequency (%) calculated for each OTU within each plot and shown in parentheses

**Table 3:** Isolation frequencies of endophytic fungal OTUs (operational taxonomic units) (indicating the relative frequency, %, in brackets) in relation to the plots and tissues from which they were recovered, Palencia, northern Spain

OTUs	Genbank Acc no.	Best hit (Genbank Acc no.)	qcovs	evalue	Plot 1	Plot 2	Plot 3	Plot 4	Plot 5	Plot 6	Plot 7	Plot 8	Total
FunID25	PV856018	*Trichoderma* sp1 (EU280068)	97	0			2						2 (0.25)
FunID23	PV856016	*Penicillium* sp. (HQ442346)	100	0								1 (2.08)	1 (0.13)
FunID116	PV856109	*Alternaria* sp. (KC584231)	98	0	1 (1.30)					2 (1.96)		2 (4.17)	5 (0.63)
FunID81	PV856074	*Fusarium* sp6 (AB587028)	88	0	27 (35.06)	35 (21.34)	6 (18.18)	18 (15.38)	23 (22.77)	21 (20.59)	26 (16.46)	2 (4.17)	158 (19.75)
FunID70	PV856063	*Fusarium* sp7 (MF120477)	100	0	9 (11.69)	19 (11.59)	2 (6.06)	25 (21.37)	5 (4.95)	10 (9.80)	6 (3.80)		76 (9.50)
FunID32	PV856025	*Fusarium* sp8 (AB587028)	91	0	19 (24.68)	71 (43.29)	7 (21.21)	46 (39.32)	53 (52.48)	47 (46.08)	30 (18.99)	10 (20.83)	283 (35.38)
FunID51	PV856044	*Fusarium* sp9 (AB510592)	97	0	1 (1.30)				3 (2.97)				4 (0.50)
FunID105	PV856098	*Fusarium* sp10 (HG422167)	96	0		2 (1.22)					1 (0.63)		3 (0.38)
FunID91	PV856084	*Hypocreales* sp. (GQ505688)	100	0		2 (1.22)					16 (10.13)		18 (2.25)
FunID95	PV856088	*Fusarium* sp1 (UDB02227335)	94	0	17 (22.08)	33 (20.12)	13 (39.39)	11 (9.40)	6 (5.94)	2 (1.96)	64 (40.51)	1 (2.08)	147 (18.38)
FunID98	PV856091	*Fusarium* sp2 (GQ505754)	99	0	3 (3.90)	2 (1.22)	3 (9.09)	17 (14.53)	8 (7.92)	3 (2.94)	13 (8.23)	27 (56.25)	76 (9.50)
FunID82	PV856075	*Fusarium* sp3 (MN954339)	100	0					2 (1.98)				2 (0.25)
FunID39	PV856032	*Fusarium* sp4 (AB586992)	98	0						10 (9.80)		4 (8.33)	14 (1.75)
FunID61	PV856054	*Cladosporium* sp. (EF679363)	100	0						5 (4.90)		1 (2.08)	6 (0.75)
FunID3	PV855996	*Fusarium* sp5 (GQ505688)	100	0					1 (0.99)	1 (0.98)	2 (1.27)		4 (0.50)
FunID50	PV856043	*Trichoderma* sp2 (LC100015)	100	0						1 (0.98)			1 (0.13)
Total					77	164	33	117	101	102	158	48	800

Note: Values indicate the number of isolates, with relative frequency (%) calculated for each OTU within each plot and shown in parentheses

### Diversity and community analyses

Microbial abundance data for each plot were organized and standardized in RStudio (R version 4.5.1) (R Core Team, 2025), with data handling performed using dplyr (Wickham et al. 2023). Relative abundance was calculated to present community composition, and genus-level distributions were visualized using pie charts. Subsequently, based on the species matrix, we computed α-diversity metrics, including Species richness (*S*), Margalef richness index (*d*), the Shannon diversity index (*H*), Simpson diversity index (1 - *D*) and Pielou’s evenness index (*J*), using functions in the vegan package (Oksanen et al. 2025). Pielou’s evenness was calculated as $\:J=\frac{H}{ln\left(S\right)}$.

A three-step approach was used to assess the relative influence of soil properties and climatic variables on endophytic bacterial and fungal communities in pea roots (Prieto-Recio et al. 2015). To avoid using highly correlated environmental variables in subsequent analyses, principal component analysis (PCA) was first conducted on standardized soil and climate variables to extract primary environmental gradients. PCA was performed in R (version 4.5.1) (R Core Team, 2025), using the vegan package (Oksanen et al. 2025).

Subsequently, the broken-stick method from the ‘BiodiversityR’ package was employed to select significant PCA axes, whereby those explaining variance above the broken-stick threshold were deemed significant (Kindt and Coe 2005; Kindt 2025). Based on this, the environmental variable most strongly associated with each significant axis was selected according to its loading magnitude, for subsequent ordination analyses.

Finally, non-metric multidimensional scaling (NMDS) was performed on the continuous environmental variables selected in step two, combined with categorical variables not included in PCA by the vegan package (Oksanen et al. 2025). This explored differences in pea root endophytic bacterial and fungal community structures across plots and their relationships with environmental factors. NMDS employed Bray–Curtis dissimilarity as the distance metric, with ordination results obtained via the metaMDS function in the vegan package. Subsequently, significant environmental variables were fitted and overlaid onto the NMDS ordination space: environmental factors were modelled using the envfit function, with significance determined via 999 permutations (*p* < 0.05). Significant categorical variables were displayed as mean points (centroids) at each factor level; significant continuous variables were overlaid as vectors onto the ordination plot, thereby indicating their relationship with the variation gradient of the endophytic microorganism community.

## Results

### Abundance and composition of endophytic bacteria and fungi

**Fig. 1:**
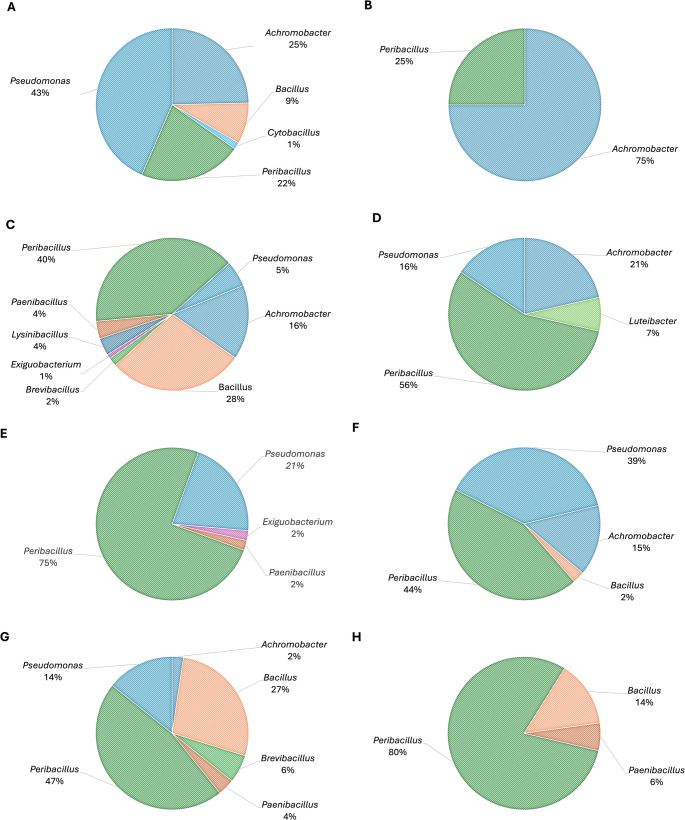
Genus-level composition of endophytic bacterial communities across various plots (**A**-**H** correspond to Plots 1–8)

**Fig. 2:**
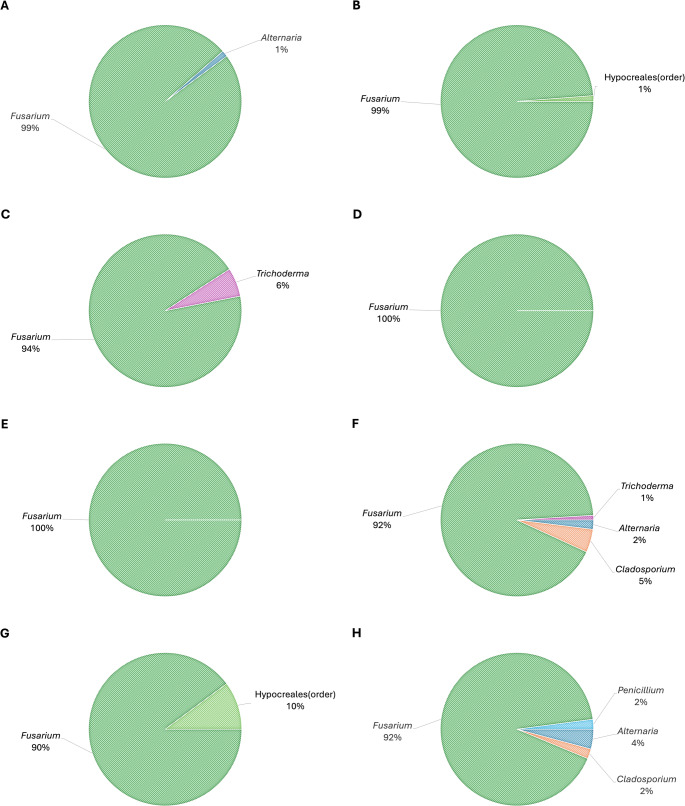
Genus-level composition of endophytic fungal communities across various plots (**A**-**H** correspond to Plots 1–8)

The genus-level composition of endophytic microorganism communities differed markedly between bacteria and fungi. Bacterial communities showed a relatively rich and variable taxonomic structure across plots, while fungal communities were consistently dominated by a single genus (Figs. 1 and 2). Regarding endophytic bacteria, multiple bacterial genera were detected in each plot, ranging from 2 to 8 in number, though the dominant genera varied between plots (Fig. 1). Certain genera exhibited high abundance across all plots, such as *Pseudomonas*, *Achromobacter*, *Peribacillus* and *Bacillus*, which were major groups in the eight plots (Fig. 1A-H). Among these, *Achromobacter* exhibited the highest relative abundance in Plot 2, accounting for approximately three-quarters of bacterial sequences in that sample (Fig. 1B); *Pseudomonas* constituted about 43% in Plot 1 (Fig. 1A); and *Peribacillus* exceeded half the abundance in Plots 4, 5, and 8 (Fig. 1D, E and H). In contrast, certain bacterial genera appeared only in isolated plots with low abundance (all below 10%), demonstrating a pattern of localized distribution. For instance, *Luteibacter*, *Lysinibacillus*, *Exiguobacterium*, *Brevibacillus* and *Paenibacillus* were detected only in Plot 3, Plot 5, or Plot 7, with abundances below 10% in each case (Fig. 1C and E, and 1G).

The genus-level composition of the endophytic fungal communities was markedly more uniform. Across all plots, *Fusarium* was the dominant genus (Fig. 2). In all plots, *Fusarium* sequences accounted for over 90% of the total, with certain plots being almost entirely composed of this genus (relative abundance approaching 99%–100%) (Fig. 2D and E). Apart from *Fusarium*, other fungal genera exhibited only low abundances. Trace amounts of other fungal genera, such as *Trichoderma*, *Alternaria*, *Cladosporium* and *Penicillium*, were detected in a few plots. However, the relative abundance of these genera typically ranged only from approximately 1% to 6%, and each was confined to occurrence in a single plot (Fig. 2A, C, F and H). Moreover, a small number of unidentified Hypocreales fungal sequences at the order level were detected in individual plots, with a maximum relative abundance of approximately 10% (Fig. 2B and G). Overall, compared to the endophytic bacteria, the endophytic fungal community exhibited lower diversity at the genus level, presenting a simple community structure dominated by a single dominant genus (primarily *Fusarium*).

### Alpha diversity of endophytic bacterial and fungal communities

**Fig. 3:**
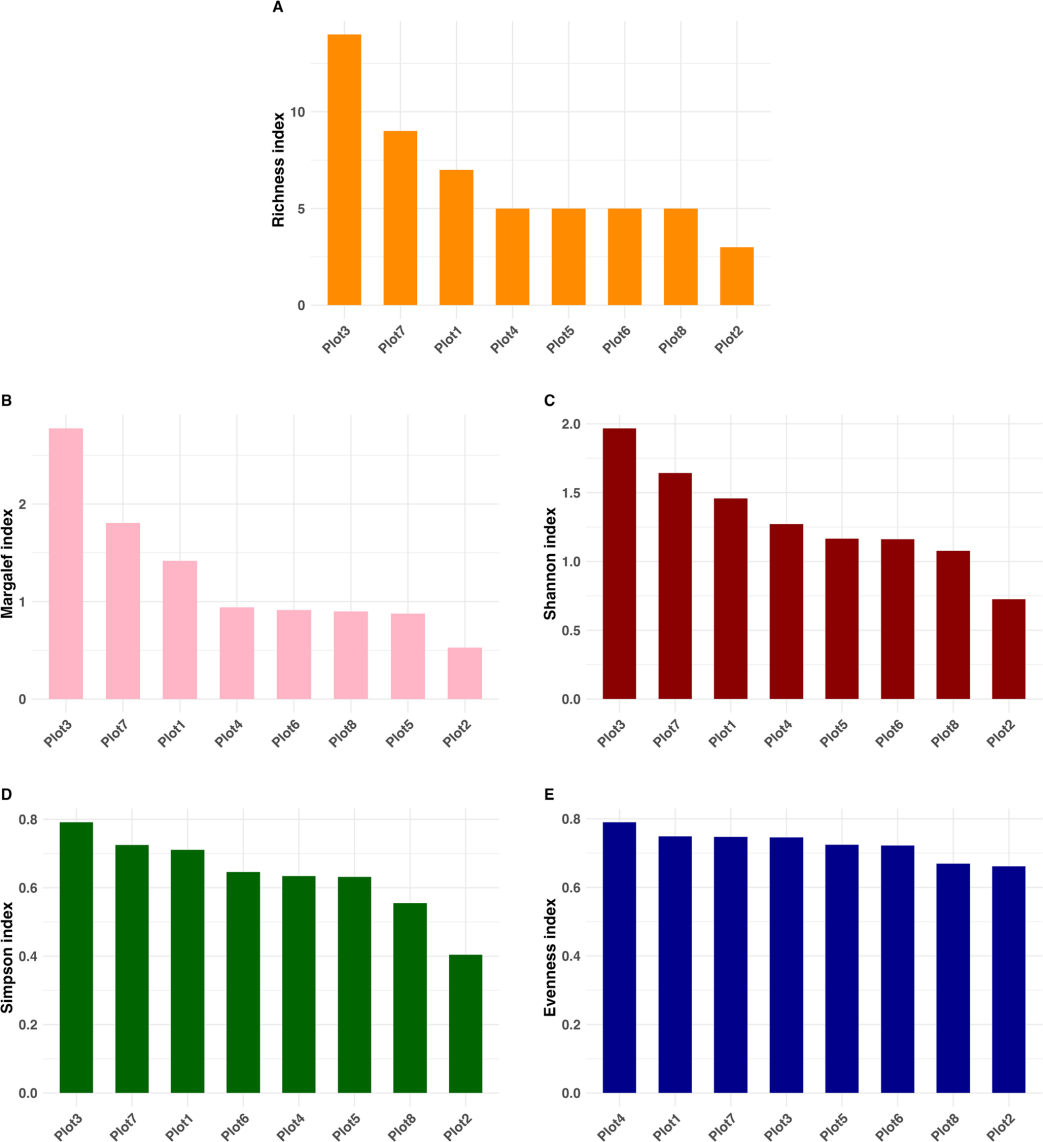
Distribution of α-diversity indices (S, d, H, 1-D, J) for endophytic bacterial communities across eight plots (**A**–**E**)

**Fig. 4:**
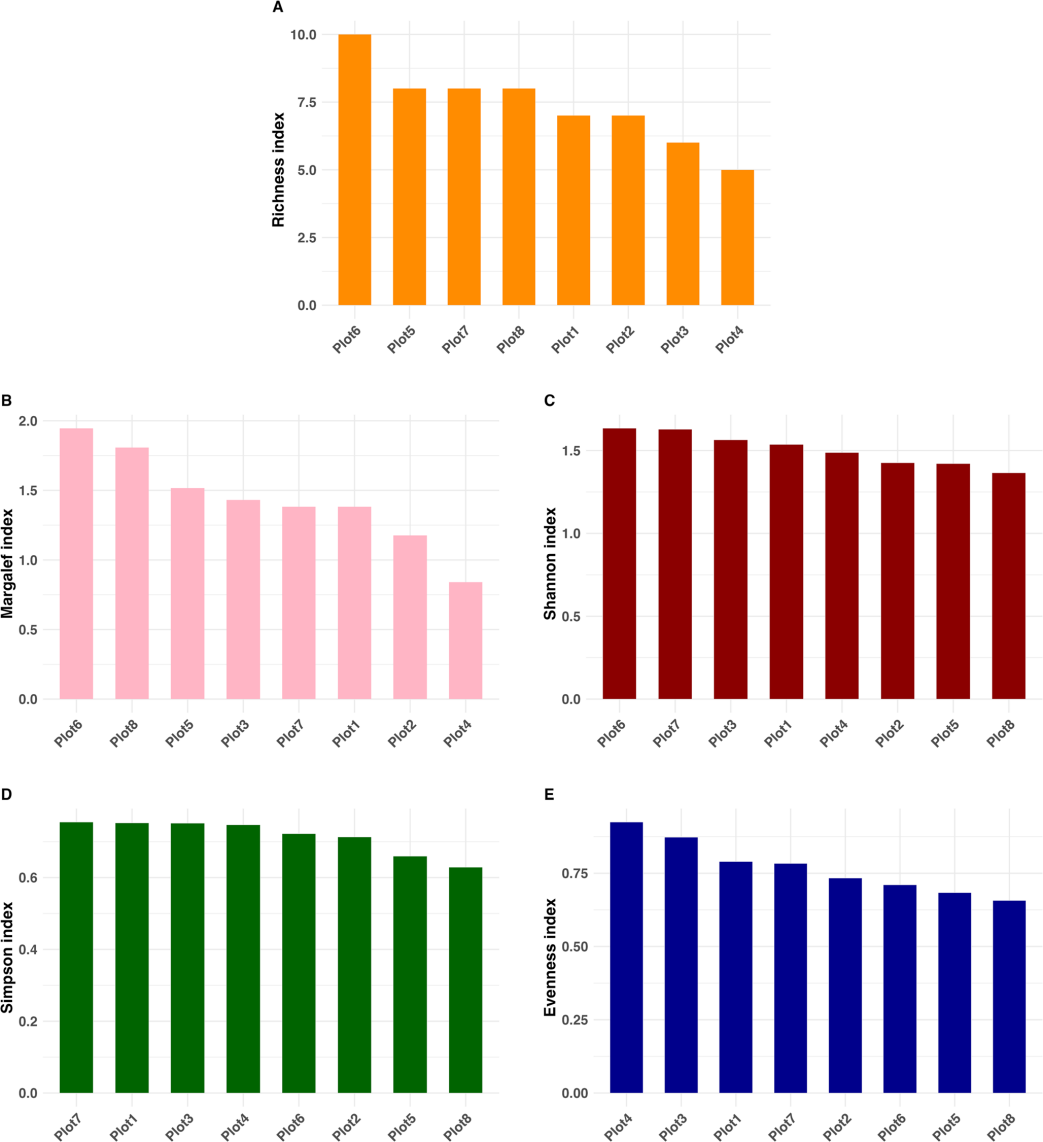
Distribution of α-diversity indices (S, d, H, 1-D, J) for endophytic fungal communities across eight plots (**A**–**E**)

Across the eight plots, α-diversity indices (*S*, *d*, *H*, 1-*D*, *J*) reveal the endophytic bacterial and fungal communities each exhibited distinct diversity patterns, with bacterial communities showing greater variability in diversity among plots than the relatively uniform fungal communities (Figs. 3 and 4).

The α-diversity indices of bacterial communities across plots exhibited marked differences. The species richness (*S* value) ranged from 3 to 14, with Plot 3 recording the highest (*S* = 14) and Plot 2 the lowest (*S* = 3) (Fig. 3A). Similarly, the Margalef index *d* peaked in Plot 3 (~ 2.78) and was lowest in Plot 2 (~ 0.53), mirroring the trend observed for *S* (Fig. 3B). The *H* ranged from 0.726 to 1.967, with Plot 3 exhibiting the highest value (*H* = 1.967) and Plot 2 the lowest (*H* = 0.726) (Fig. 3C). Similarly, the Simpson index 1-*D* peaked in Plot 3 (~ 0.792) and was lowest in Plot 2 (~ 0.404) (Fig. 3D). This indicated that Plot 3 had the highest bacterial community diversity, characterized by species richness and even distribution. In contrast, Plot 2 exhibited extremely low species numbers, dominated by a few dominant bacteria, and thus the lowest diversity. *H* and 1-*D* values for other plots fell between these extremes, reflecting moderate diversity. The evenness index J was generally high and similar across plots (approximately 0.72–0.75). Plot 4 exhibited the highest *J* value (0.790), while Plot 2 had the lowest (0.661), further indicating that abundance distribution was relatively balanced across most bacterial communities except Plot 2 (Fig. 3E). Overall, species richness and α-diversity appeared to vary among plots, with Plot 3 displaying the highest α-diversity and Plot 2 the lowest.

The α-diversity of fungal communities also varied between plots, though to a lesser extent than that of bacteria (Fig. 4). Fungal OTU richness S ranged from 5 to 10, with Plot 6 exhibiting the highest value (S = 10) and Plot 4 the lowest (S = 5) (Fig. 4A). Similarly, the Margalef index *d* reached its maximum (~ 1.95) at Plot 6 and its minimum (~ 0.84) at Plot 4 (Fig. 4B). The Shannon diversity index *H* showed a narrower range (1.36–1.63), with relatively similar diversity levels across sites. Plot 6 exhibited the highest *H* value (*H* = 1.634), while Plot 8 had the lowest (*H* = 1.364) (Fig. 4C). Notably, Plot 4 with the lowest richness (*S* = 5) did not exhibit the lowest *H* value (*H* = 1.487), owing to its high evenness (*J* = 0.924) (Fig. 4A, C and E). Several fungal species showed balanced abundance distribution, thereby maintaining elevated diversity. Conversely, Plot 8 exhibited moderate richness (*S* = 8) but the lowest evenness among all plots (*J* = 0.656), with certain fungal species dominating markedly, resulting in lower diversity (*H* = 1.364) (Fig. 4A, C and E). The Simpson index 1-*D* ranged from 0.628 to 0.754 across fungal communities, with limited variation between plots: the highest value occurred in Plot 7 (1-*D* = 0.754) and the lowest in Plot 8 (1-*D* = 0.628), consistent with the trends (Fig. 4D). Overall, fungal endophytic communities exhibited some variation in richness and diversity across plots, though to a lesser extent than bacteria. Most sites displayed moderate levels of fungal α-diversity.

### PCA-based environmental variables selection and NMDS ordination of endophytic communities

To select variables for NMDS analysis, a principal component analysis (PCA) was conducted. The breakpoint method indicated that the PCA for soil properties retained two principal component axes (explaining approximately 84% cumulative variance), while the PCA for climate variables also retained two principal component axes (explaining approximately 94% cumulative variance) (Table 4). Representative variables selected from these retained PCA axes are as follows: for soil properties, silt content and exchangeable magnesium; for climate variables, spring mean temperature and annual mean precipitation (Supplementary Table 3).

**Table 4:** Selection of significant axes from principal component analyses by the broken-stick method

	Axes						
	1	2	3	4	5	6	7
soil properties							
eigenvalue	6.467835	3.662039	0.9086723	0.4887102	0.3873722	0.08005847	5.31E-03
percentage of variance	**53.898624**	**30.516988**	7.5722688	4.0725847	3.2281013	0.66715393	4.43E-02
broken-stick percentage	**37.040816**	**22.755102**	15.6122449	10.8503401	7.2789116	4.42176871	2.04E + 00
climatic variables							
eigenvalue	13.88239	10.53813	1.011386	0.5523365	0.01521498	4.15E-04	1.32E-04
percentage of variance	**53.39379**	**40.53127**	3.889946	2.124371	0.05851914	1.60E-03	5.09E-04
broken-stick percentage	**37.04082**	**22.7551**	15.612245	10.8503401	7.27891156	4.42E + 00	2.04E + 00

Note: PCA axes with larger percentages of variance than broken-stick variances are significant (in bold type)

**Fig. 5:**
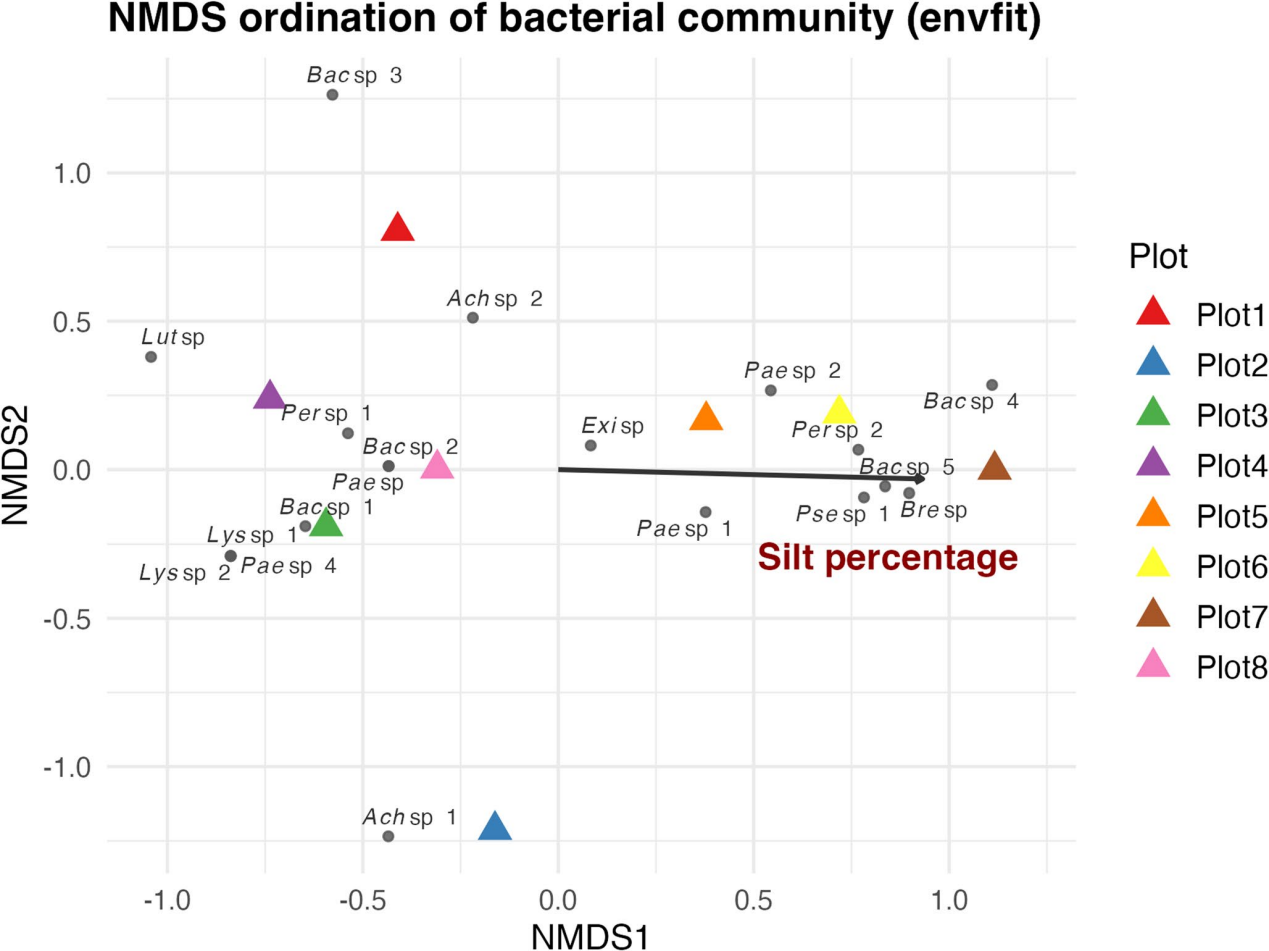
Non-metric multidimensional scaling (NMDS) of endophytic bacterial communities based on Bray–Curtis distance, with plots, OTUs (labelled with genus name + sp number, e.g. *Ach* sp1) and significant environmental factors (silt percentage)

**Fig. 6:**
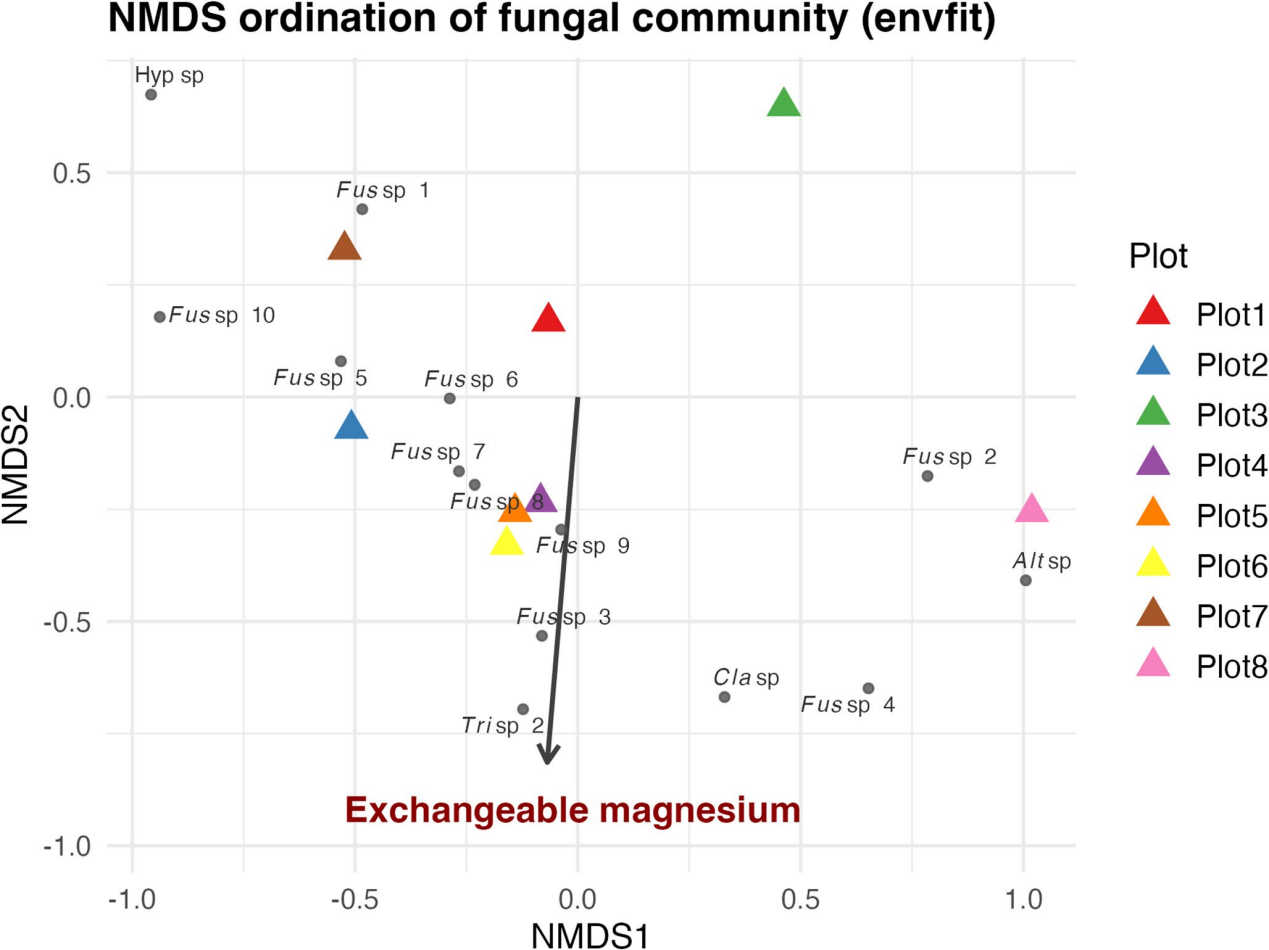
Non-metric multidimensional scaling (NMDS) of endophytic fungal communities based on Bray–Curtis distance with plots, OTUs (labelled with genus name + sp. number, e.g. *Fus* sp1) and significant environmental factors (exchangeable magnesium)

In the NMDS ordination analysis, soil silt percentage showed a significant correlation with bacterial community distribution (r² = 0.87, *P* = 0.007); while exchangeable magnesium, spring mean temperature, annual precipitation and categorical variables all failed to reach significance (*P* > 0.05) (Supplementary Table 4). The first axis (horizontal axis) of the bacterial community NMDS ordination primarily reflected the silt content gradient, with sites of higher silt content clustering at one end of this axis, indicating distinct bacterial community compositions in soils with higher silt proportions (Fig. 5). For fungal communities, exchangeable magnesium showed a significant correlation with NMDS ordination (r² = 0.67, *P* = 0.046), while silt content, spring mean temperature, annual precipitation and categorical variables exerted no significant influence (*P* > 0.05) (Supplementary Table 4). The first NMDS axis for endophytic fungal communities primarily correlated with the exchangeable magnesium gradient. Sites with higher exchangeable magnesium content clustered at one end of this axis, indicating that increased exchangeable magnesium content in soil corresponded with distinct differences in fungal community composition (Fig. 6).

## Discussion

This study characterized the composition and diversity of cultivable endophytic bacteria and fungi associated with pea roots under field conditions. Despite the limitations of culture-dependent isolation, we repeatedly isolated a highly overlapping set of endophytic taxa across different plots, suggesting the presence of a relatively stable cultivable fraction within healthy pea roots. Among the recurrently isolated taxa, bacterial genera such as *Bacillus*, *Paenibacillus*, *Pseudomonas*, *Lysobacter* and *Achromobacter* were frequently detected, while fungal genera also included isolates of *Fusarium* and *Alternaria*. These findings are highly consistent with recent high-throughput sequencing studies on endophytes in peas and other leguminous crops. Previous studies on pea endophytes based on amplicon sequencing identified genera such as *Rhizobium*, *Pseudomonas*, and *Bacillus* as common members of root and nodule-associated bacterial communities (Chibeba et al. 2020; Hnini et al. 2023). Studies in peas and other legumes have likewise frequently reported *Bacillus*, *Pseudomonas* and *Paenibacillus* among recurrent endophytic bacterial genera (Lv et al. 2021; Mayhood and Mirza 2021; Martínez-Hidalgo et al. 2022). Taken together, these comparisons suggest that several bacterial taxa recovered here overlap with commonly reported legume-associated endophytes, while still representing only the cultivable subset of the pea root endophytic microbiome. 

In the fungal fraction, *Fusarium* and *Alternaria* were among the most frequently isolated genera. This pattern has also been reported in some culture-based studies of root-associated endophytic fungi (Abeywickrama et al. 2023). The root endosphere is generally considered a more selective niche than surrounding soil or the rhizosphere, often showing lower diversity and stronger host filtering, which may favor the repeated detection of a limited number of successful colonizers (Sun et al. 2021; Guo et al. 2024a; Wang et al. 2026). As a result, the high recovery frequency of *Fusarium* in this study is better interpreted as a feature of the culturable fungal fraction rather than as a direct representation of the overall fungal endophytic community in pea roots. More broadly, the repeated recovery of a limited number of bacterial and fungal genera in this study may reflect both their persistence in plant-associated habitats and their higher recoverability under the culture conditions used.

Compared with some studies based on metagenomic or amplicon sequencing, the α diversity indices obtained in this study were relatively low. Considering our research strategy of culture-dependent methods combined with selecting one or two representative colonies per morphotype for identification, this discrepancy is more likely to reflect methodological limitations rather than genuinely low diversity of endophytic microorganisms within pea roots in the study area. Multiple comparative studies in recent years examining culture-dependent versus culture-independent methods have highlighted that traditional culturing techniques capture only a small fraction of the endosymbiotic microbial community, favoring the rapid-growing and easily cultivable “cultivable subset”, thereby significantly underestimating true diversity (Anguita-Maeso et al. 2020; Zheng et al. 2021; Posada et al. 2024). Furthermore, the ‘morphotype-based representative selection’ strategy employed in this study similarly compresses richness estimates. Colony morphology is influenced by multiple factors including medium composition, incubation time, and colony density. A single morphotype may encompass multiple phylogenetic lineages, while conversely, different morphotypes may belong to the same species (Škaloud et al. 2020; Rattray et al. 2023; Duckett et al. 2024). Consequently, selecting representative colonies for identification based on morphology inevitably ‘merges’ cryptic species, leading to underestimation of species richness S and the derived Shannon index. Similar conclusions have been validated in other pooled methodology studies (Gupta et al. 2025). Nevertheless, the isolate collection generated here may still be useful for subsequent strain recovery, inoculation assays and functional screening.

PCA results indicated that, at the farmland scale examined in this study, variation in cultivable endophytic community composition was most strongly associated with two soil-related variables: silt percentage and exchangeable magnesium. Envfit analysis further revealed that silt percentage was significantly correlated with bacterial community structure, whilst exchangeable magnesium was significantly correlated with fungal community structure. These patterns are broadly consistent with previous studies showing that soil physicochemical properties can contribute to variation in root-associated microbial assemblages (Xia et al. 2020; Guo et al. 2024b). This may be explained by that soil texture influences water retention, aeration, pore structure, and nutrient dynamics in the root zone, thereby altering the conditions under which some bacterial taxa are more readily established or recovered (Islam et al. 2020; Wille et al. 2020). Likewise, exchangeable magnesium may reflect aspects of soil nutrient status and plant nutritional condition that are associated with fungal community variation (Qin et al. 2020; Tang et al. 2022; Liu et al. 2023; Hsieh et al. 2024). However, given the limited number of plots and the correlational nature of the ordination-based analyses, these results should be interpreted as environmental associations rather than as evidence of direct causal mechanisms. In this context, the observed relationships are most parsimoniously interpreted as environmental filtering acting on the cultivable fraction of the endophytic microbiota.

In contrast to soil-related variables, climate PCA indicated that annual precipitation and mean spring temperature were the main axes of climatic variation among sampling sites, yet these variables showed no significant fit to the NMDS ordination of cultivable endophytic communities. This does not necessarily indicate that climate is irrelevant to pea endophytes; rather, it suggests that no detectable climate signal emerged at the spatial scale and gradient examined here. A likely explanation is that all plots were located within the same region and therefore experienced relatively limited climatic variation compared with the broader gradients considered in regional studies or experimental climate manipulations. In addition, soil properties may provide more immediate and detectable environmental contrasts at this local scale, and the limited number of plots may have reduced statistical power to detect moderate climate effects (Bütikofer et al. 2020; Huth and Dubrovský 2021). Overall, our results suggest that, within the range of conditions sampled here, soil-related variables were more directly associated with variation in the cultivable endophytic fraction than the climatic variables considered (Deltedesco et al. 2020). This study therefore identifies a set of recurrent cultivable endophytic taxa in healthy pea roots and their preliminary associations with local environmental conditions, providing a basis for future strain-based functional studies and for more comprehensive microbiome analyses using culture-independent approaches.

## Conclusion

This study characterized the cultivable endophytic bacterial and fungal assemblages associated with field-grown healthy pea roots after nodule removal. Across plots, we repeatedly recovered a highly overlapping set of cultivable endophytic taxa, indicating the presence of a relatively stable cultivable fraction within healthy pea roots. Within this fraction, bacterial communities showed greater between-plot variation, whereas *Fusarium* and *Alternaria* were among the most frequently recovered fungal genera. At the spatial scale examined here, variation in the cultivable bacterial community was associated with silt percentage, whereas variation in the cultivable fungal community was associated with exchangeable magnesium. Overall, this study provides a culture-based overview of endophytic taxa in healthy pea roots and their preliminary associations with local soil conditions, thereby offering a basis for future strain-based functional studies and for more comprehensive microbiome analyses using culture-independent approaches.

## Supplementary Information

Below is the link to the electronic supplementary material.

Supplementary Material 1 (DOCX 17.9 KB)

Supplementary Material 2 (DOCX 22.0 KB) 

Supplementary Material 3 (DOCX 18.8 KB)

Supplementary Material 4 (DOCX 22.2 KB)

## Data Availability

The datasets generated and/or analyzed during the current study, including OTU tables and environmental variables, are available from the corresponding author on reasonable request. Representative sequences have been deposited in the NCBI GenBank database (accession numbers provided in Tables 2 and 3).
