# Supplementary material for: Unravelling the associations between local environmental factors, soil properties and cultivable root-associated endophytes in dry pea (Pisum sativum L.)
Source: World J Microbiol Biotechnol. 2026 Jun 22;42(7):366. doi: 10.1007/s11274-026-05092-9 (PMC13287291; doi:10.1007/s11274-026-05092-9)
Supplement: Supplementary file 1 — Supplementary Material 1 (DOCX 17.9 KB) [file 11274_2026_5092_MOESM1_ESM.docx]

Supplementary Table 4 Coefficients of correlation between environmental properties (soil and climatic) and the significant axes of the principal component analysis.

| **Soil properties** | | |
| --- | --- | --- |
| Parameter/PC axes | PC1 | PC2 |
| pH | 0.70177853 | -0.482943 |
| Electrical conductivity (mS cm⁻¹) | -0.77667259 | -0.3187085 |
| Sand (%) | 0.70139864 | 0.4789326 |
| Silt (%) | **-0.80411386** | -0.2073266 |
| Clay (%) | -0.57227122 | -0.5616768 |
| Oxidizable organic matter (%) | -0.66819873 | 0.5473877 |
| Available phosphorus (mg kg⁻¹) | -0.52665225 | 0.6150784 |
| Available potassium (mg kg⁻¹) | -0.79185174 | 0.1502036 |
| Exchangeable calcium (cmol_c_ kg⁻¹) | -0.69601224 | -0.4093755 |
| Exchangeable magnesium (cmol_c_ kg⁻¹) | -0.02880685 | **-0.7423686** |
| Exchangeable sodium (cmol_c_ kg⁻¹) | -0.14814324 | -0.5224285 |
| Total nitrogen (%) | -0.72113312 | 0.4267677 |
| **Climatic properties** | | |
| Parameter/PC axes | PC1 | PC2 |
| UTM coordinates X | -0.3466789 | -0.62960675 |
| UTM coordinates Y | -0.6061178 | -0.33416892 |
| Spring precipitation (mm) | -0.4183754 | -0.57797901 |
| Spring precipitation average (mm) | -0.4161379 | -0.57917105 |
| Summer precipitation (mm) | 0.2479416 | -0.6536797 |
| Summer precipitation average (mm) | 0.2472714 | -0.65346388 |
| Autumn precipitation (mm) | -0.3253723 | -0.64155987 |
| Autumn precipitation average (mm) | -0.3229641 | -0.64287341 |
| Winter precipitation (mm) | 0.164582 | -0.67601438 |
| Winter precipitation average (mm) | 0.160806 | -0.67722187 |
| Annual precipitation average (mm) | -0.1187479 | **-0.70305214** |
| Minimum average spring temperature (°C) | 0.6140839 | -0.29674639 |
| Minimum average summer temperature (°C) | 0.6124666 | -0.24164317 |
| Minimum average autumn temperature (°C) | -0.2153619 | -0.60015215 |
| Minimum average winter temperature (°C) | 0.4194053 | -0.51591338 |
| Minimum average annual temperature (°C) | 0.4194053 | -0.51591338 |
| Maximum average spring temperature (°C) | 0.7150988 | -0.04095287 |
| Maximum average summer temperature (°C) | 0.7101657 | -0.0800225 |
| Maximum average autumn temperature (°C) | 0.6964982 | 0.01727787 |
| Maximum average winter temperature (°C) | 0.6289935 | 0.09408943 |
| Maximum average annual temperature (°C) | 0.7144723 | 0.0588991 |
| Spring average temperature (°C) | **0.7182243** | -0.05259848 |
| Summer average temperature (°C) | 0.6991802 | -0.16878049 |
| Autumn average temperature (°C) | 0.6795124 | -0.21761342 |
| Winter average temperature (°C) | 0.6670704 | 0.2142451 |
| Annual average temperature (°C) | 0.70631 | -0.1308153 |

Note: The highest correlations are shown in bold type.
