# Supplementary material for: Unravelling the associations between local environmental factors, soil properties and cultivable root-associated endophytes in dry pea (Pisum sativum L.)
Source: World J Microbiol Biotechnol. 2026 Jun 22;42(7):366. doi: 10.1007/s11274-026-05092-9 (PMC13287291; doi:10.1007/s11274-026-05092-9)
Supplement: Supplementary file 2 — Supplementary Material 2 (DOCX 22.0 KB) [file 11274_2026_5092_MOESM2_ESM.docx]

Supplementary Table 1

Description of dried peas (*Pisum sativum* L.) plots and sampling locations in Palencia province, Spain, including plant phenological stages, previous crop, sowing dates, work tasks, pesticide, and fertilization.

| Sample plot number | Plot1 | Plot2 | Plot3 | Plot4 | Plot5 | Plot6 | Plot7 | Plot8 |
| --- | --- | --- | --- | --- | --- | --- | --- | --- |
| UTM coordinates X | 401435 | 401235 | 401635 | 401735 | 347656 | 348256 | 372956 | 374756 |
| UTM coordinates Y | 4661181 | 4660781 | 4660881 | 4660081 | 4642364 | 4651564 | 4644964 | 4644464 |
| Number of phenological stage to which the sampled plants belong (counts)* | R1×2,  R2×3,  R3×7 | V3×1,  V7×1,  R1×2,  R2×7,  R3×1 | V6×1,  R1×1,  R2×1,  R3×8,  R4×1 | R1×3,  R2×3,  R3×6 | R1×4,  R2×2,  R3×5,  R4×1 | V4×1,  V5×1,  V7×1,  V17×1,  R1×1,  R2×2,  R3×4,  R4×1 | V8×1,  R1×2,  R3×1,  R4×7,  R5×1 | R1×3,  R3×2,  R4×6,  R5×1 |
| Previous crop | Barley | | | | | | | |
| Sowing date | 02/02/2024 | | | | 25/01/2024 | | 03/02/2024 | 04/02/2024 |
| Work tasks | Surface work:  harrowing and conventional sowing with jet seed drills | | | | Direct seeding:  with no tillage and no-till seed drills | | Intermediate work:  22nd December 2023 mini-chisel; 30th January 2024 disc harrow; 1st February 2024 disc roller; 4th February 2024 disc harrow.  Sowing with jet seed drills | Intermediate work:  14th December 2023: Grid pass.  31st January 2024: Second pass of Harrow.  1st January 2024: Passing of water roller.  5th February 2024: Passage of water roller.  14th March 2024: Water roller passed due to wild boar damage.  Sowing with jet seed drills |
| Pesticide | Yes:  Summer: glyphosate.  At sowing: pendimethalin and prosulfocarb.  Spring: clethodim. | | | | Yes:  Pre-sowing:glyphosate.  Post-emergence: broadleaf herbicide | | Yes:  7th February 2024 herbicide (1.25 L Bismak with 1.5 L Roundup Ultraplus with 200 L water).  10th April 2024 herbicide (1.75 L Quilop).  13th May 2024 insecticide Aphox (200 g with 500 ml of wetting agent). | Yes:  28th July 2023 herbicide (2.6 L Roundup Ultraplus with 110 L water)； 10th April 2024 herbicide (1.75 L Quilop with 190 L water)； 13th May 2024: Insecticide Aphox (200 g with 500 ml of wetting agent) |
| Fertilized | Have not been fertilized | | | | Have not been fertilized | | Yes:  10th April 2024 fertilizer 2.8L Bombardier, 3.2 L Landamine BMO with 190L water. | |
| *V1(First node): first unfolded stipule, clasping the main stem; V2 to Vn: Second stipule unfolded, third, fourth, etc.; R1(Flower bud): Flower bud present at one or more nodes; R2(Beginning bloom): Flower open at one or more nodes; R3(Flat pod): Flat pod at one or more nodes; R4(Full pod): Green seeds fill the pod cavity at one or more nodes; R5(Beginning maturity): Leaves and lower pods start to turn yellow (<https://www.manitobapulse.ca/2018/10/field-pea-growth-staging-guide/>). | | | | | | | | |
