# Supplementary material for: Unravelling the associations between local environmental factors, soil properties and cultivable root-associated endophytes in dry pea (Pisum sativum L.)
Source: World J Microbiol Biotechnol. 2026 Jun 22;42(7):366. doi: 10.1007/s11274-026-05092-9 (PMC13287291; doi:10.1007/s11274-026-05092-9)
Supplement: Supplementary file 3 — Supplementary Material 3 (DOCX 18.8 KB) [file 11274_2026_5092_MOESM3_ESM.docx]

Supplementary Table 4 Environmental variable fitting results (envfit) for NMDS ordination of endophytic communities (both Bacterial and Fungal)

| **Bacterial communities** | | | | | | | |
| --- | --- | --- | --- | --- | --- | --- | --- |
| Environmental variables | | | NMDS1 | NMDS2 | r^2^ | Pr(>r) |  |
| Silt (%) | | | 0.99942 | -0.03401 | 0.8695 | 0.007 | ** |
| Exchangeable magnesium (cmol_c_ kg⁻¹) | | | 0.92368 | 0.38316 | 0.0615 | 0.868 |  |
| Spring average temperature (°C) | | | 0.98873 | 0.14973 | 0.1545 | 0.665 |  |
| Annual precipitation average (mm) | | | -0.94039 | -0.3401 | 0.4384 | 0.239 |  |
| Sowing date | | | 0.37535 | 0.92688 | 0.2854 | 0.384 |  |
| Work tasks | Direct seeding | | -0.1501 | -0.2923 | 0.1544 | 0.789 |  |
|  | Intermediate work | | 0.2477 | 0.0362 |  |  |  |
|  | Surface work | | -0.0488 | 0.128 |  |  |  |
| Herbicide | Herbicide yes | | 0.000 | 0.000 | 0.000 | 0.000 |  |
| Insecticide | Insecticide no | | -0.0826 | -0.0121 | 0.062 | 0.853 |  |
|  | Insecticide yes | | 0.2477 | 0.0362 |  |  |  |
| Fertilizer | Fertilizer no | | -0.0826 | -0.0121 | 0.062 | 0.853 |  |
|  | Fertilizer yes | | 0.2477 | 0.0362 |  |  |  |
| **Fungal communities** | | | | | | |  |
| Environmental variables | | | NMDS1 | NMDS2 | r^2^ | Pr(>r) |  |
| Silt (%) | | | -0.7368 | -0.67611 | 0.1192 | 0.699 |  |
| Exchangeable magnesium (cmol_c_ kg⁻¹) | | | -0.08399 | -0.99647 | 0.6678 | 0.046 | * |
| Spring average temperature (°C) | | | 0.88614 | -0.46343 | 0.1998 | 0.584 |  |
| Annual precipitation average (mm) | | | 0.16189 | 0.98681 | 0.2921 | 0.397 |  |
| Sowing date | | | 0.37535 | 0.92688 | 0.2854 | 0.384 |  |
| Work tasks | | Direct seeding | -0.1501 | -0.2923 | 0.1544 | 0.789 |  |
|  |  | Intermediate work | 0.2477 | 0.0362 |  |  |  |
|  |  | Surface work | -0.0488 | 0.128 |  |  |  |
| Herbicide | | Herbicide yes | 0 | 0 | 0.000 | 0.000 |  |
| Insecticide | | Insecticide no | -0.0826 | -0.0121 | 0.062 | 0.853 |  |
|  |  | Insecticide yes | 0.2477 | 0.0362 |  |  |  |
| Fertilizer | | Fertilizer no | -0.0826 | -0.0121 | 0.062 | 0.853 |  |
|  |  | Fertilizer yes | 0.2477 | 0.0362 |  |  |  |

Note: Pr(>r) denotes the *P*-value associated with the correlation coefficient r. **P*<0.05, ***P*<0.01, ****P*<0.001.
