# Supplementary material for: Unravelling the associations between local environmental factors, soil properties and cultivable root-associated endophytes in dry pea (Pisum sativum L.)
Source: World J Microbiol Biotechnol. 2026 Jun 22;42(7):366. doi: 10.1007/s11274-026-05092-9 (PMC13287291; doi:10.1007/s11274-026-05092-9)
Supplement: Supplementary file 4 — Supplementary Material 4 (DOCX 22.2 KB) [file 11274_2026_5092_MOESM4_ESM.docx]

Supplementary Table 2 Summary of environmental properties (soil and climatic) of 8 sampling plots. Mean ± standard error (SE)

| **Soil properties** | | | | | | | | |
| --- | --- | --- | --- | --- | --- | --- | --- | --- |
| Parameter/Plot | plot1 | plot2 | plot3 | plot4 | plot5 | plot6 | plot7 | plot8 |
| pH | 8.545 ± 0.045 | 8.625 ± 0.005 | 8.39 ± 0.03 | 8.35 ± 0.02 | 8.525 ± 0.005 | 8.515 ± 0.015 | 7.915 ± 0.225 | 8.405 ± 0.045 |
| Electrical conductivity (mS cm⁻¹) | 0.06 ± 0 | 0.07 ± 0 | 0.055 ± 0.005 | 0.085 ± 0.005 | 0.095 ± 0.005 | 0.115 ± 0.005 | 0.125 ± 0.005 | 0.105 ± 0.005 |
| Sand (%) | 68.84 ± 0 | 63.34 ± 0.5 | 74.84 ± 0 | 63.34 ± 0.5 | 49.62 ± 0.5 | 36.12 ± 1 | 40.34 ± 0.5 | 64.84 ± 1 |
| Silt (%) | 10.28 ± 0 | 12.78 ± 0.5 | 8.28 ± 0 | 9.28 ± 0 | 15.78 ± 0.5 | 17.78 ± 0.5 | 23.28 ± 1 | 15.78 ± 0.5 |
| Clay (%) | 20.88 ± 0 | 23.88 ± 0 | 16.88 ± 0 | 27.38 ± 0.5 | 34.6 ± 0 | 46.1 ± 0.5 | 36.38 ± 0.5 | 19.38 ± 0.5 |
| Soil texture | Sandy clay loam | Sandy clay loam | Sandy clay loam | Sandy clay | Coarse clay | Fine clay | Coarse clay | Sandy clay loam |
| Oxidizable organic matter (%) | 1.325 ± 0.045 | 1.21 ± 0 | 1.925 ± 0.065 | 1.395 ± 0.015 | 0.99 ± 0.1 | 1.175 ± 0.055 | 3.83 ± 0.05 | 1.42 ± 0.05 |
| Available phosphorus (mg kg⁻¹) | 21.25 ± 0.95 | 13.1 ± 5.3 | 22.8 ± 1.9 | 32.35 ± 1.35 | 4.8 ± 0.8 | 6 ± 0.2 | 60.65 ± 4.15 | 37.35 ± 1.35 |
| Available potassium (mg kg⁻¹) | 132.5 ± 4.5 | 113 ± 1 | 213 ± 5 | 210 ± 6 | 169.5 ± 0.5 | 266 ± 1 | 369 ± 6 | 247 ± 4 |
| Exchangeable calcium (cmol_c_ kg⁻¹) | 22.5 ± 1.2 | 32.75 ± 0.25 | 16 ± 0.6 | 20.2 ± 0.1 | 36.95 ± 0.05 | 42.3 ± 0 | 45.9 ± 0.6 | 34.85 ± 0.15 |
| Exchangeable magnesium (cmol_c_ kg⁻¹) | 0.58 ± 0.02 | 1.02 ± 0.01 | 0.525 ± 0.005 | 1.515 ± 0.005 | 2.21 ± 0.01 | 1.795 ± 0.015 | 0.65 ± 0.02 | 1.135 ± 0.025 |
| Exchangeable sodium (cmol_c_ kg⁻¹) | 0.055 ± 0.005 | 0.045 ± 0.005 | 0.035 ± 0.005 | 0.04 ± 0 | 0.035 ± 0.005 | 0.1 ± 0.03 | 0.04 ± 0.01 | 0.08 ± 0.03 |
| Total nitrogen (%) | 0.110 ± 0 | 0.1 ± 0 | 0.14 ± 0 | 0.115 ± 0.005 | 0.105 ± 0.005 | 0.12 ± 0 | 0.275 ± 0.005 | 0.095 ± 0.045 |
| **Climatic properties** | | | | | | | | |
| Parameter/Plot | plot1 | plot2 | plot3 | plot4 | plot5 | plot6 | plot7 | plot8 |
| Spring precipitation (mm) | 128.5 | 128.3 | 128.5 | 128.8 | 115.1 | 110.4 | 118.2 | 117.8 |
| Spring precipitation average (mm) | 42.8 ± 5.9 | 42.8 ± 5.9 | 42.8 ± 5.9 | 42.9 ± 5.9 | 38.4 ± 6.2 | 36.8 ± 7.5 | 39.4 ± 7.6 | 39.3 ± 7.2 |
| Summer precipitation (mm) | 81.6 | 81.7 | 81.5 | 81.8 | 71.4 | 69.7 | 86.6 | 86.4 |
| Summer precipitation average (mm) | 27.2 ± 7.7 | 27.2 ± 7.7 | 27.2 ± 7.7 | 27.3 ± 7.7 | 23.8 ± 6.6 | 23.2 ± 6.0 | 28.9 ± 7.0 | 28.8 ± 7.1 |
| Autumn precipitation (mm) | 130.6 | 130.7 | 130.7 | 130.9 | 115.2 | 112.1 | 121.7 | 122.6 |
| Autumn precipitation average (mm) | 43.5 ± 4.1 | 43.6 ± 4.1 | 43.6 ± 4.1 | 43.6 ± 4.1 | 38.4 ± 4.9 | 37.4 ± 4.9 | 40.6 ± 4.6 | 40.9 ± 4.7 |
| Winter precipitation (mm) | 129.1 | 128.9 | 129.1 | 129.2 | 120.6 | 113.6 | 132.1 | 131.8 |
| Winter precipitation average (mm) | 43.0 ± 4.0 | 43.0 ± 4.0 | 43.0 ± 4.0 | 43.1 ± 4.0 | 40.2 ± 4.1 | 37.9 ± 4.4 | 44.0 ± 6.0 | 43.9 ± 5.8 |
| Annual precipitation average (mm) | 469.8 ± 3.2 | 469.6 ± 3.2 | 469.8 ± 3.2 | 470.7 ± 3.2 | 422.3 ± 3.1 | 405.8 ± 3.1 | 458.6 ± 3.2 | 458.6 ± 3.2 |
| Minimum average spring temperature (°C) | 3.8 ± 1.4 | 3.8 ± 1.5 | 3.8 ± 1.5 | 3.8 ± 1.5 | 3.7 ± 1.5 | 3.8 ± 1.5 | 4.0 ± 1.5 | 4.2 ± 1.5 |
| Minimum average summer temperature (°C) | 11.5 ± 0.8 | 11.5 ± 0.8 | 11.5 ± 0.8 | 11.5 ± 0.8 | 11.6 ± 0.8 | 11.4 ± 0.8 | 11.6 ± 0.7 | 11.8 ± 0.7 |
| Minimum average autumn temperature (°C) | 6.2 ± 2.1 | 6.2 ± 2.1 | 6.2 ± 2.1 | 6.2 ± 2.1 | 6.1 ± 2.1 | 6.1 ± 2.1 | 6.1 ± 2.2 | 6.2 ± 2.2 |
| Minimum average winter temperature (°C) | 0.0 ± 0.3 | 0.0 ± 0.3 | 0.0 ± 0.3 | 0.0 ± 0.3 | -0.1 ± 0.3 | -0.1 ± 0.3 | 0.0 ± 0.3 | 0.2 ± 0.2 |
| Minimum average annual temperature (°C) | 5.4 ± 1.4 | 5.4 ± 1.4 | 5.4 ± 1.4 | 5.4 ± 1.4 | 5.3 ± 1.4 | 5.3 ± 1.4 | 5.4 ± 1.4 | 5.6 ± 1.4 |
| Maximum average spring temperature (°C) | 16.0 ± 1.8 | 16.0 ± 1.8 | 16.0 ± 1.8 | 16.0 ± 1.8 | 16.3 ± 1.8 | 16.2 ± 1.7 | 16.9 ± 1.8 | 17.1 ± 1.9 |
| Maximum average summer temperature (°C) | 27.1 ± 1.4 | 27.1 ± 1.4 | 27.1 ± 1.4 | 27.1 ± 1.4 | 27.4 ± 1.4 | 27.2 ± 1.4 | 27.7 ± 1.7 | 28.2 ± 1.3 |
| Maximum average autumn temperature (°C) | 18.3 ± 3.5 | 18.3 ± 3.5 | 18.3 ± 3.5 | 18.4 ± 3.6 | 18.5 ± 3.5 | 18.5 ± 3.5 | 18.9 ± 3.6 | 18.9 ± 3.6 |
| Maximum average winter temperature (°C) | 9.1 ± 0.6 | 9.1 ± 0.6 | 9.1 ± 0.6 | 9.1 ± 0.6 | 9.2 ± 0.7 | 9.1 ± 0.7 | 9.2 ± 0.8 | 9.2 ± 0.8 |
| Maximum average annual temperature (°C) | 17.6 ± 2.1 | 17.6 ± 2.1 | 17.6 ± 2.1 | 17.6 ± 2.1 | 17.9 ± 2.2 | 17.8 ± 2.1 | 18.2 ± 2.2 | 18.3 ± 2.2 |
| Spring average temperature (°C) | 9.9 ± 1.1 | 9.9 ± 1.1 | 9.9 ± 1.1 | 9.9 ± 1.1 | 10.1 ± 1.1 | 10.0 ± 1.1 | 10.4 ± 1.2 | 10.6 ± 1.2 |
| Summer average temperature (°C) | 19.3 ± 0.7 | 19.3 ± 0.7 | 19.3 ± 0.7 | 19.3 ± 0.8 | 19.4 ± 0.8 | 19.3 ± 0.8 | 19.8 ± 0.7 | 20.0 ± 0.7 |
| Autumn average temperature (°C) | 12.3 ± 2.0 | 12.3 ± 2.0 | 12.3 ± 2.0 | 12.3 ± 2.0 | 12.3 ± 2.0 | 12.3 ± 2.0 | 12.5 ± 2.0 | 12.6 ± 2.1 |
| Winter average temperature (°C) | 4.5 ± 0.3 | 4.5 ± 0.3 | 4.5 ± 0.3 | 4.5 ± 0.3 | 4.6 ± 0.3 | 4.6 ± 0.3 | 4.6 ± 0.3 | 4.7 ± 0.3 |
| Annual average temperature (°C) | 11.5 ± 1.8 | 11.5 ± 1.8 | 11.5 ± 1.8 | 11.5 ± 1.8 | 11.6 ± 1.8 | 11.5 ± 1.8 | 11.8 ± 1.8 | 11.9 ± 1.8 |
